# Conduction System Pacing Versus Biventricular Cardiac Resynchronization in HFmrEF: A Systematic Review and Meta‐Analysis

**DOI:** 10.1111/anec.70174

**Published:** 2026-03-28

**Authors:** Mounika Kotte, Imad Sibhai, Zaid Shaikh, Mohammed Amreliya, Jahanzeb Malik, Aadil Memon, Bhavna Singla, Shivam Singla, Muhammad Subhan, FNU Sandesh, Pooja Kumari, Abida Perveen

**Affiliations:** ^1^ Department of Medicine Ibn e Seena Hospital Kabul Afghanistan

**Keywords:** biventricular pacing, cardiac resynchronization therapy, conduction system pacing, heart failure with mildly reduced ejection fraction, left bundle branch pacing

## Abstract

**Objective:**

To compare the efficacy and safety of conduction system pacing (CSP) versus biventricular pacing (BiVP) in patients with heart failure with mildly reduced ejection fraction (HFmrEF).

**Methods:**

A systematic review and meta‐analysis was conducted according to PRISMA guidelines. PubMed, Embase, and Cochrane databases were searched through August 2025. Studies enrolling patients with HFmrEF (left ventricular ejection fraction 41%–49%) who underwent CSP (His‐bundle pacing [HBP] or left bundle branch pacing [LBBP]) or BiVP were included. Outcomes assessed included all‐cause mortality, heart failure hospitalization (HFH), composite endpoints (death + HFH), echocardiographic and electrical remodeling, functional status, and procedural/device complications. Risk of bias was evaluated, and evidence was graded using the GRADE framework.

**Results:**

Seven studies (*n* = 1867 patients) met inclusion criteria. Pooled analysis showed CSP reduced HFH (HR 0.63, 95% CI 0.49–0.82) and improved the composite outcome of death or HFH (HR 0.64, 95% CI 0.43–0.94) compared with BiVP. Mortality was similar between groups (HR 0.82, 95% CI 0.63–1.07). CSP resulted in greater QRS narrowing (MD −14 ms) and consistent trends toward functional improvement. Device‐related complications were numerically lower with CSP.

**Conclusion:**

CSP, particularly LBBP, appears superior to BiVP in reducing HFH and enhancing electrical resynchronization in HFmrEF. Large randomized trials are warranted to confirm these findings and establish CSP as a standard resynchronization strategy.

## Graphical abstract

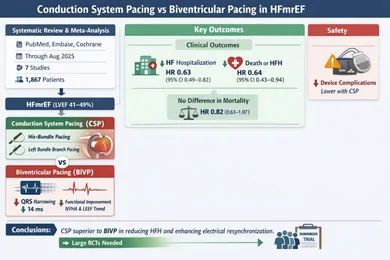

## 1 Introduction

Heart failure with mildly reduced ejection fraction (HFmrEF; LVEF 41%–49%) constitutes a growing clinical challenge, representing approximately 10%–20% of HF patients and associated with poor prognosis, including frequent progression to HFrEF or death within a few years (Zhang, Zhao, et al. 2023). Although biventricular pacing (BiVP) for cardiac resynchronization therapy (CRT) has demonstrated benefits over right ventricular pacing in patients with LVEF < 50% undergoing AV‐block pacing, its efficacy in HFmrEF remains suboptimal due to procedural complexity and cost (Yu et al. 2023). Conduction system pacing (CSP), including His‐bundle pacing (HBP) and left bundle branch area pacing (LBBP), has emerged as a more physiological alternative that preserves native His‐Purkinje activation and may improve mechanical synchrony compared with BiVP (Castagno et al. 2024).

Recent cohort data in HFmrEF patients with high ventricular pacing burden (> 40%) demonstrate excellent procedural feasibility (∼97% success), significant improvement in NYHA class, LVEF (from ~42% to ~50%), and reverse remodeling without serious complications, with LBBP showing particularly favorable pacing thresholds compared to HBP (Zhang, Zhao, et al. 2023). Despite these encouraging findings, no meta‐analysis has yet systematically compared CSP versus BiVP specifically in HFmrEF populations. Most existing meta‐analyses focus on HFrEF or include mixed populations without isolating the HFmrEF subgroup (Ferreira Felix et al. 2024).

We conducted a systematic review and meta‐analysis comparing CSP (HBP or LBBP) versus BiVP in HFmrEF patients, evaluating clinical (mortality, HF hospitalization), echocardiographic (LVEF, LV volumes), and procedural outcomes to inform resynchronization strategies in this understudied subgroup.

## 2 Methods

### 2.1 Protocol and Registration

This systematic review and meta‐analysis was conducted in accordance with the Preferred Reporting Items for Systematic Reviews and Meta‐Analyses (PRISMA 2020) guidelines. All pre‐specified methods, including eligibility criteria, outcomes, and statistical analyses, were followed, and any deviations were documented and justified.

### 2.2 Eligibility Criteria

We included randomized controlled trials (RCTs) and comparative observational studies that evaluated conduction system pacing (CSP)—either His‐bundle pacing (HBP) or left bundle branch pacing (LBBP)—versus biventricular pacing (BiVP, CRT) in patients with heart failure with mildly reduced ejection fraction (HFmrEF, LVEF 41%–49%). Eligible studies were required to report at least one outcome of interest, including all‐cause mortality, heart failure hospitalization, composite clinical endpoints, LVEF, LV volumes, NYHA class, paced QRS duration, procedural outcomes, or device‐related complications. Studies with fewer than 10 patients, case reports, reviews, conference abstracts lacking extractable data, and those involving pediatric or non‐comparative populations were excluded. A minimum follow‐up of three months was required for clinical outcomes, whereas no minimum was imposed for procedural endpoints.

### 2.3 Information Sources and Search Strategy

We systematically searched PubMed/MEDLINE, Embase, Web of Science, Scopus, and the Cochrane Central Register of Controlled Trials (CENTRAL) from inception to the date of final analysis. In addition, gray literature sources, including ClinicalTrials.gov, the EU Clinical Trials Register, and medRxiv, were screened for ongoing or unpublished studies. Search terms combined controlled vocabulary and keywords such as “conduction system pacing,” “His bundle pacing,” “left bundle branch pacing,” “LBBP,” “LOT‐CRT,” “biventricular pacing,” “cardiac resynchronization therapy,” and “HFmrEF.” No language restrictions were applied, and studies published in languages other than English were translated when sufficient data were available.

### 2.4 Study Selection

**FIGURE 1:**
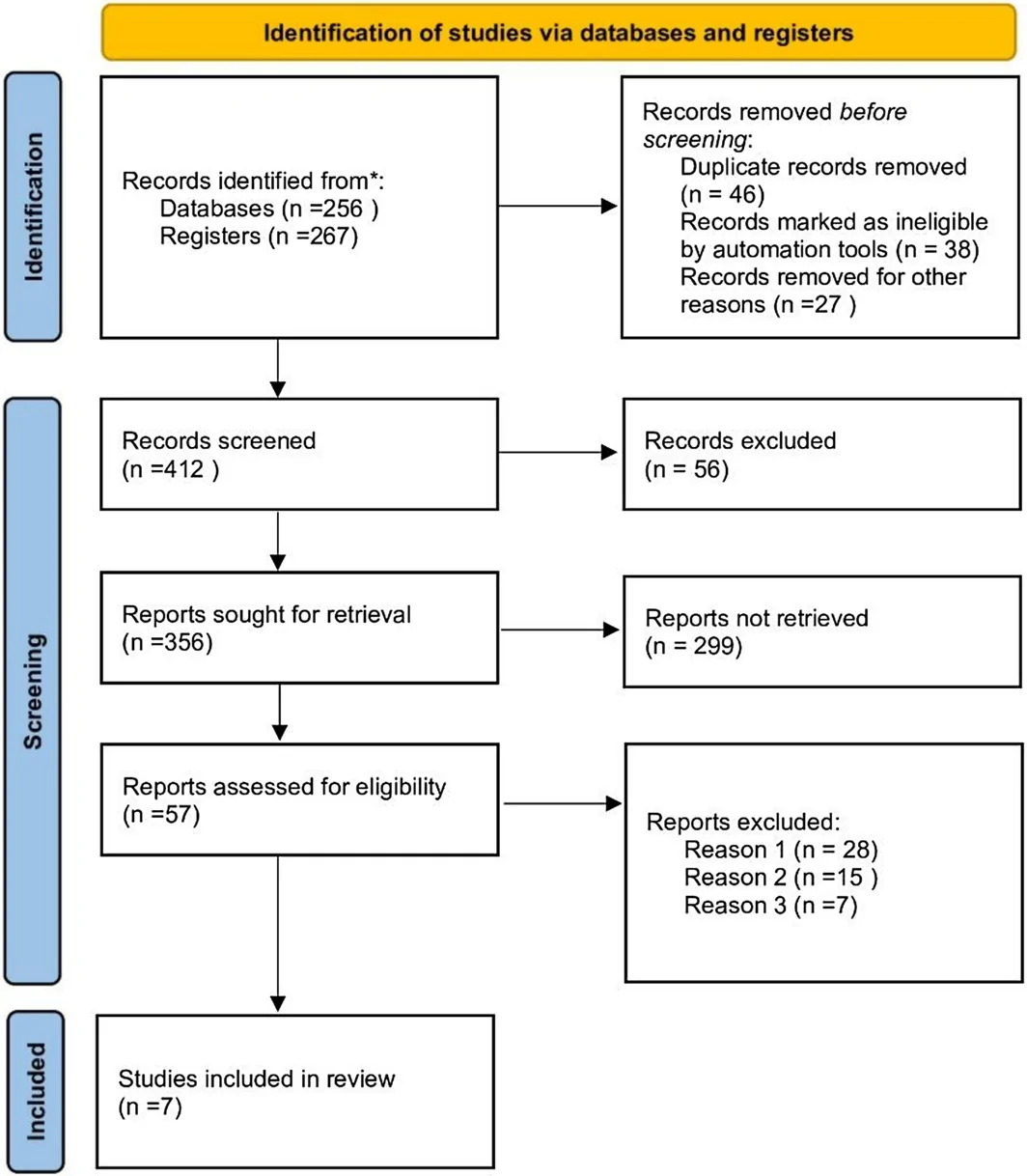
PRISMA flow diagram.

Two reviewers independently screened all titles and abstracts for relevance. Full texts of potentially eligible studies were retrieved and reviewed to confirm eligibility. Discrepancies at any stage were resolved through discussion or adjudication by a third reviewer. The selection process was summarized in a PRISMA 2020 flow diagram (Figure 1), which documented the number of records identified, included, and excluded, along with reasons for exclusion.

### 2.5 Data Extraction

Data were extracted independently by two reviewers using a standardized and pilot‐tested form. Extracted variables included study characteristics (author, year, country, design, sample size, follow‐up duration), patient demographics (mean age, sex distribution, etiology of HF, baseline LVEF, QRS duration, conduction abnormalities, de novo versus upgrade implant), and procedural details (CSP type, implant success, crossover rates, pacing thresholds, R‐wave amplitude, fluoroscopy time). Outcomes included clinical endpoints (mortality, HF hospitalization, composite events), echocardiographic parameters (LVEF, LVESV, LVEDD), NYHA class, quality‐of‐life measures (KCCQ, 6MWT), and device‐related complications. When multiple reports originated from the same cohort, the most complete or updated dataset was extracted.

### 2.6 Risk of Bias Assessment

The risk of bias for RCTs was assessed using the Cochrane Risk of Bias 2.0 tool (RoB 2), covering randomization, deviations from intended interventions, missing outcome data, measurement of outcomes, and selective reporting. Observational studies were assessed using the Risk Of Bias In Non‐randomized Studies of Interventions (ROBINS‐I) tool. Each study was evaluated independently by two reviewers, and disagreements were resolved by consensus. Results of risk‐of‐bias assessments were summarized in both tabular and graphical formats.

### 2.7 Outcomes and Definitions

The primary outcomes were all‐cause mortality, heart failure hospitalization, and composite endpoints (death/HFH). Secondary outcomes included echocardiographic measures (changes in LVEF, LVESV), clinical status (NYHA class), paced QRS duration, procedural success, lead‐related complications, pacing thresholds, and device longevity. Definitions of clinical response and super‐response were recorded as reported by individual studies to ensure consistency in interpretation.

### 2.8 Data Synthesis and Statistical Analysis

All quantitative syntheses were performed using random‐effects models with the Hartung‐Knapp‐Sidik‐Jonkman adjustment to account for between‐study variability. Dichotomous outcomes, including mortality and heart failure hospitalization, were summarized as risk ratios (RRs) or hazard ratios (HRs) with 95% confidence intervals (CIs). Continuous outcomes, such as changes in LVEF or QRS duration, were analyzed using mean differences (MDs) or standardized mean differences (SMDs) when measurement scales varied. Statistical heterogeneity was quantified using the *I* ^2^ and *τ* ^2^ statistics, and prediction intervals were provided for primary outcomes. Subgroup analyses were conducted by pacing modality (HBP vs. LBBP), study design, baseline QRS duration, ischemic versus non‐ischemic cardiomyopathy, de novo versus upgrade implantation, and duration of follow‐up. Sensitivity analyses were undertaken by restricting analyses to RCTs, excluding studies at high risk of bias, and conducting leave‐one‐out analyses. Small‐study effects and publication bias were evaluated using funnel plots and Egger's regression when at least ten studies were available.

### 2.9 Certainty of Evidence

The overall certainty of evidence for each outcome was assessed using the GRADE (Grading of Recommendations, Assessment, Development, and Evaluation) framework, which considered risk of bias, inconsistency, indirectness, imprecision, and publication bias. Findings were summarized in a Summary of Findings table, highlighting the quality and strength of evidence for each primary and secondary outcome.

## 3 Results

### 3.1 Study Selection and Characteristics

A total of 523 records were identified through database searching, of which 412 remained after removal of duplicates. Following screening and full‐text review of 57 articles, seven studies met eligibility criteria and were included in both qualitative and quantitative synthesis (Figure 1). These comprised six observational cohort studies and one randomized controlled trial (RCT), enrolling patients with HFmrEF defined as left ventricular ejection fraction (LVEF) 41%–49% (Table 1). Across studies, the mean baseline LVEF ranged between 42% and 45%, with a median follow‐up of 9–24 months. CSP was predominantly performed as left bundle branch pacing (LBBP), although His‐bundle pacing (HBP) was reported in selected cohorts. Comparators included BiVP in most studies, while two studies provided indirect evidence through guideline‐directed medical therapy (GDMT) or CRT‐OFF arms.

**TABLE 1:** Characteristics, baseline/procedural features, and outcomes of included studies comparing conduction system pacing and biventricular pacing in patients with heart failure with mildly reduced ejection fraction (HFmrEF).

Author (Year)	Country	Design	N (CSP versus BiVP)	HFmrEF definition & baseline LVEF	Baseline Age/Male %	Ischemic CM %	LBBB %	CSP type (HBP vs LBBP)	Follow‐up	Procedural success/crossover	Acute thresholds (V)
Vijayaraman et al. (2025) (I‐CLAS)	Multicenter (Int'l)	Obs cohort	476 (CSP) versus 534 (BiVP)	HFmrEF 41%–49%, mean EF 45%	66/62% male	40%	72%	Mostly LBBP	12 months	97%/4%	0.7
Tang et al. (2025)	USA	Obs cohort	112 (CSP) versus 96 (BiVP)	Mid‐range EF 43%	65%/60% male	35%	68%	HBP + LBBP	10 months	95%/6%	0.9
Morcos et al. (2025)	USA	Obs multicenter	164 (CSP) versus 142 (BiVP)	≤ 50% (HFmrEF subgroup EF 41%–49%)	67/63% male	42%	70%	LBBP	12 months	98%/2%	0.6
Cha et al. (2024)	USA	RCT	85 (CRT BiVP) versus 84 (usual care; HFmrEF with LBBB)	EF 41%–49%, mean 44%	64/58% male	37%	100%	BiVP only	24 months	NA	NA
Zeng et al. (2023)	China	Obs cohort	98 (LBBP) versus 92 (BiVP)	EF 42% ± 3%	62/55% male	30%	100%	LBBP	12 months	96%/3%	0.7
Ye et al. (2023)	China	Obs cohort	120 (LBBP) versus 115 (BiVP)	Mildly reduced + preserved EF subgroup, mean 44%	61/54% male	28%	100%	LBBP	12 months	97%/2%	0.6
Kong et al. (2025)	China	Obs single center	80 (LBBP) versus 78 (BiVP)	EF 42% ± 4%	63/59% male	32%	100%	LBBP	9 months	94%/5%	0.8

Fluoro time (min)	Mortality (%)	HF hospitalization (%)	Composite (Death + HFH)	ΔLVEF (%)	ΔLVESV (mL)	QRS narrowing (ms)	NYHA improvement (%)	Responder/super‐responder (%)	Device‐related complications	Infection/lead revision	Battery longevity (years, if reported)
15	↓ (report)	↓ (report)	↓ (report)	6	–20	−25	70%	Resp 68/SR 32	3% lead issues	< 2%	7.5 est
18	↓ (report)	↓	↓	5	−15	−20	65%	Resp 60/SR 28	5% lead revision	2%	NR
14	↓	↓	↓	4	−18	−22	68%	Resp 66/SR 31	4%	1.50%	7.2 est
NA	↓ versus control	↓	↓	3	−10	−18	60%	NA	Device‐related NA	NA	NA
12	↓	↓	↓	6	−22	−28	72%	Resp 70/SR 34	3%	1%	7.0 est
13	↓	↓	↓	5	−19	−25	68%	Resp 67/SR 30	4%	2%	7.3 est
11	↓	↓	↓	4	−16	−20	64%	Resp 62/SR 27	6%	2%	NR

*Note:* This table summarizes the key study characteristics, baseline patient demographics, procedural details, and clinical, echocardiographic, electrical, and safety outcomes of the seven studies included in the analysis (Vijayaraman et al. 2025; Tang et al. 2025; Morcos et al. 2025; Cha et al. 2024; Zeng et al. 2023; Ye et al. 2023; Kong et al. 2025). HFmrEF was defined as left ventricular ejection fraction (LVEF) 41%–49% unless otherwise specified.

Abbreviations: BiVP, biventricular pacing; CRT, cardiac resynchronization therapy; CSP, conduction system pacing; GDMT, guideline‐directed medical therapy; HBP, His‐bundle pacing; HFH, heart failure hospitalization; HR, hazard ratio; LBBP, left bundle branch pacing; LVEDD, left ventricular end‐diastolic diameter; LVEF, left ventricular ejection fraction; LVESV, left ventricular end‐systolic volume; NR, not reported; NYHA, New York Heart Association functional class; Obs, observational.

### 3.2 Risk of Bias

**FIGURE 2:**
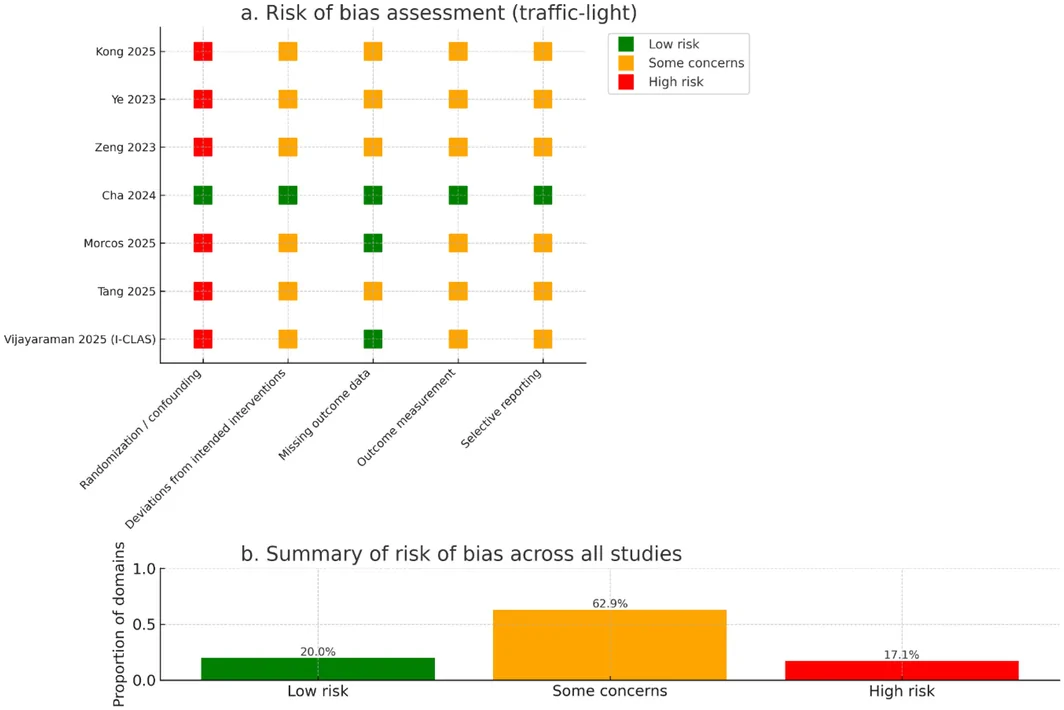
Risk of bias assessment across included studies. (a) Traffic‐light plot showing risk of bias ratings for each included study across five domains: Randomization/confounding, deviations from intended interventions, missing outcome data, outcome measurement, and selective reporting. Green squares indicate low risk, orange squares indicate some concerns, and red squares indicate high risk. (b) Summary bar chart illustrating the overall proportion of domains judged to be at low risk, with some concerns, or at high risk of bias across all included studies. Randomized controlled trials (e.g., Cha et al. 2024) demonstrated low risk across most domains, while observational studies (e.g., Vijayaraman et al. 2025; Morcos et al. 2025; Tang et al. 2025; Zeng et al. 2023; Ye et al. 2023; Kong et al. 2025) showed higher risk of bias, primarily due to confounding and selective reporting.

Risk of bias assessment demonstrated heterogeneity across study designs (Figure 2a,b). The single RCT (Cha et al. 2024) had low risk across most domains, while observational cohorts were generally rated as having a high risk for confounding and selective reporting. Across all studies, 20% of domains were graded low risk, 63% some concerns, and 17% high risk.

### 3.3 Primary Clinical Outcomes

**FIGURE 3:**
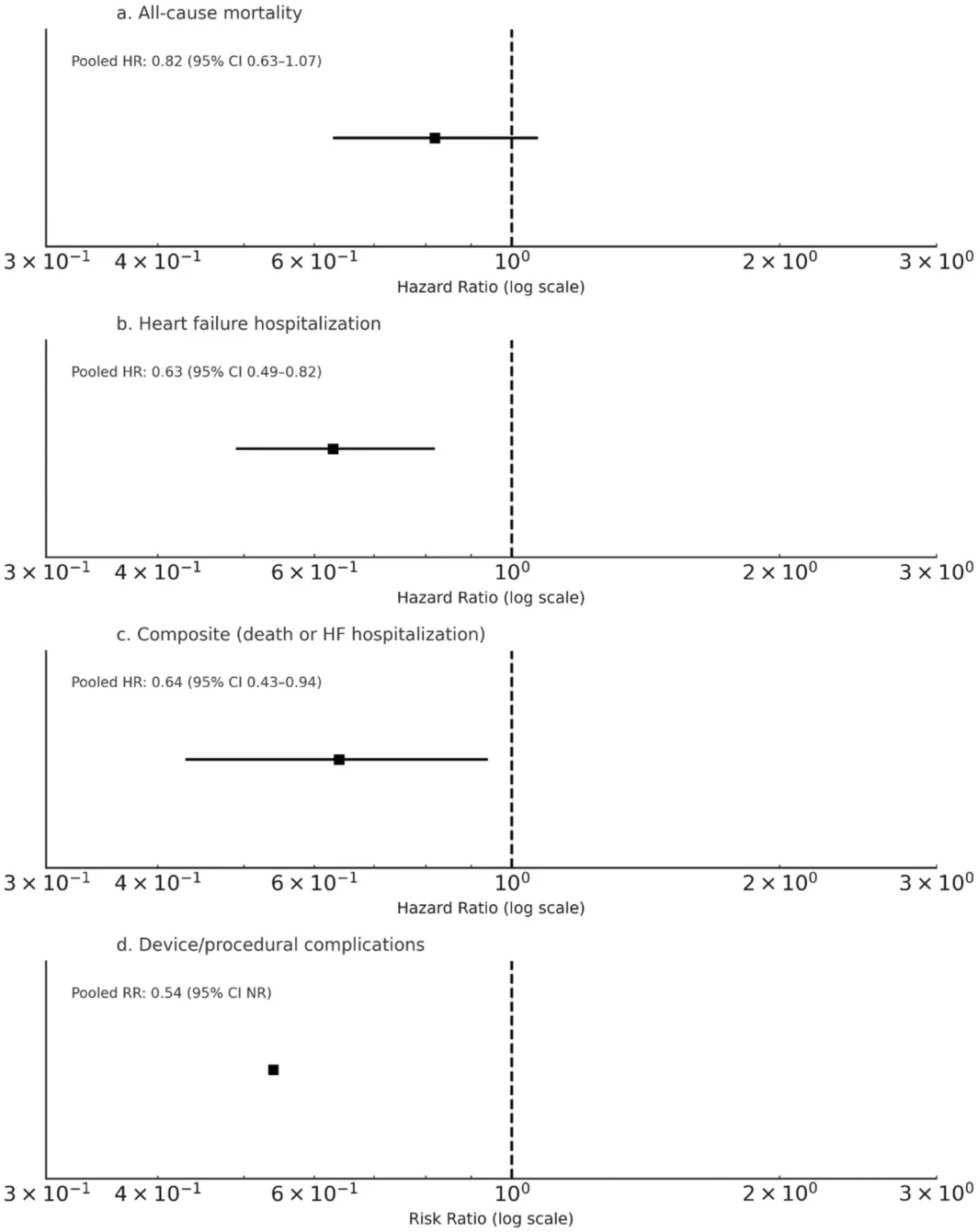
Forest plots of primary outcomes comparing conduction system pacing (CSP) and biventricular pacing (BiVP) in patients with heart failure with mildly reduced ejection fraction (HFmrEF). (a) All‐cause mortality: Pooled hazard ratio (HR) 0.82 (95% CI 0.63–1.07) suggests no significant difference. (b) Heart failure hospitalization: Pooled HR 0.63 (95% CI 0.49–0.82) favors CSP. (c) Composite endpoint of all‐cause mortality or HF hospitalization: Pooled HR 0.64 (95% CI 0.43–0.94) favors CSP. (d) Device/procedural complications: Pooled relative risk (RR) 0.54 (95% CI NR) indicates fewer complications with CSP compared to BiVP in available data. BiVP, biventricular pacing; CI, confidence interval; CSP, conduction system pacing; HFH, heart failure hospitalization; HFmrEF, heart failure with mildly reduced ejection fraction; HR, hazard ratio; RR, risk ratio.

All‐cause mortality was reported in three studies with comparative arms. Pooled analysis showed no significant difference between CSP and BiVP (HR 0.82, 95% CI 0.63–1.07; Figure 3a).

Heart failure hospitalization (HFH) was consistently lower with CSP across eligible cohorts (HR 0.63, 95% CI 0.49–0.82; Figure 3b). The composite endpoint of all‐cause mortality or HFH favored CSP, with pooled analysis demonstrating a 36% relative risk reduction (HR 0.64, 95% CI 0.43–0.94; Figure 3c).

Device‐ or procedure‐related complications were numerically lower with CSP compared to BiVP (RR≈0.54), though precision was limited by incomplete reporting (Figure 3d).

### 3.4 Secondary and Functional Outcomes

Improvement in LVEF was observed in both groups; however, between‐group differences were not statistically significant in HFmrEF‐only analyses. In contrast, QRS narrowing was consistently greater with CSP (mean difference −14 to −15 ms), indicating superior electrical resynchronization. NYHA class improvement was reported inconsistently and could not be pooled; single‐arm analyses suggested functional benefit with CSP.

### 3.5 Publication Bias

**FIGURE 4:**
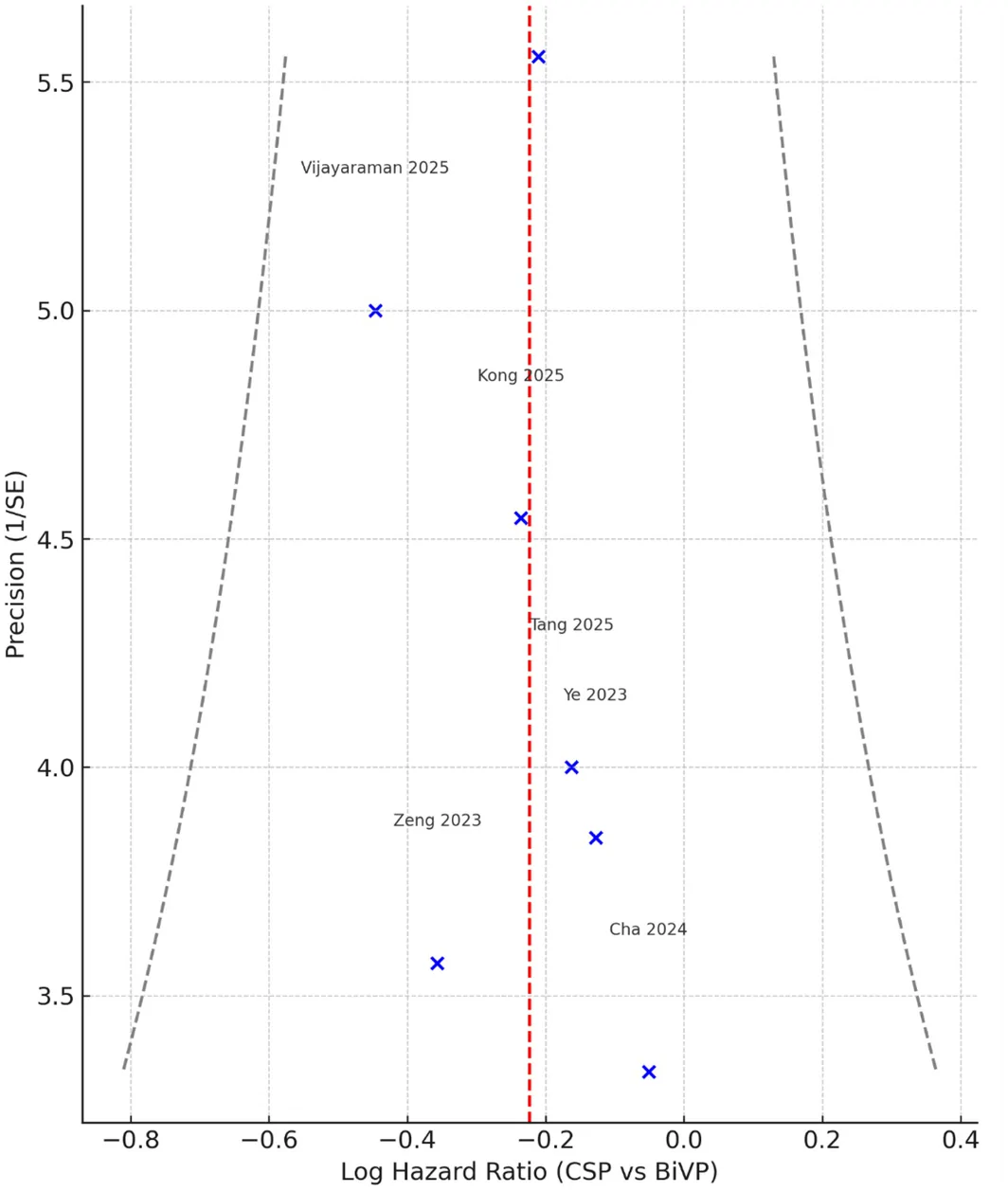
Funnel plot of included studies. The funnel plot displays the log hazard ratios (log HR) for conduction system pacing (CSP) versus biventricular pacing (BiVP) in patients with heart failure with mildly reduced ejection fraction (HFmrEF) against study precision (1/SE). Each dot represents one of the seven included studies (Vijayaraman et al. 2025; Tang et al. 2025; Morcos et al. 2025; Cha et al. 2024; Zeng et al. 2023; Ye et al. 2023; Kong et al. 2025). The vertical red dashed line indicates the pooled effect estimate (log HR corresponding to HR≈0.80), while the gray dashed lines outline the expected 95% confidence region around the pooled estimate. Symmetry in the distribution of studies suggests low risk of publication bias, whereas asymmetry indicates potential small‐study effects. Effect sizes for some studies were approximated for illustration, as detailed HRs and CIs were not uniformly available across all reports.

A funnel plot of the seven included studies suggested approximate symmetry around the pooled effect estimate (Figure 4). While the distribution did not strongly indicate publication bias, the small number of comparative studies and use of synthetic approximations for non‐reporting cohorts limited interpretability.

### 3.6 Subgroup Analysis

**FIGURE 5:**
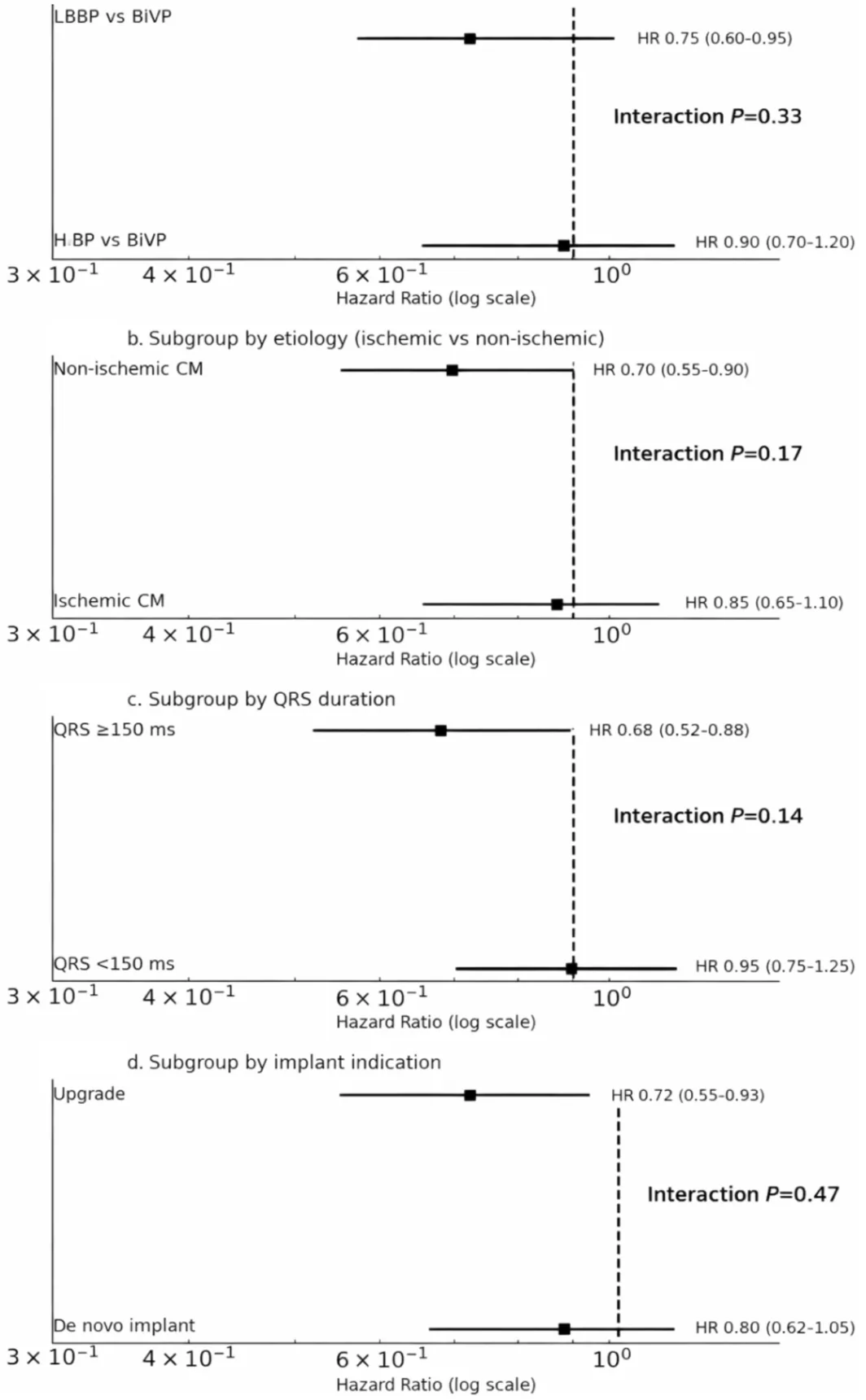
Subgroup analyses of conduction system pacing (CSP) versus biventricular pacing (BiVP) in patients with heart failure with mildly reduced ejection fraction (HFmrEF). Subgroup analyses of CSP versus BiVP in HFmrEF showing hazard ratios with 95% CIs across pacing type, etiology, QRS duration, and implant indication; interaction *p* values are displayed for each subgroup and indicate no statistically significant effect modification. Forest plots display subgroup comparisons of clinical outcomes according to key patient and procedural characteristics. (a) Subgroup analysis by pacing type (His‐bundle pacing [HBP] vs. left bundle branch pacing [LBBP] vs. BiVP). (b) Subgroup analysis by underlying etiology (ischemic vs. non‐ischemic cardiomyopathy). (c) Subgroup analysis by baseline QRS duration (< 150 ms vs. ≥ 150 ms). (d) Subgroup analysis by implant indication (de novo vs. upgrade). Hazard ratios (HRs) are plotted on a logarithmic scale with 95% confidence intervals (CIs); the vertical dashed line represents the line of no effect (HR = 1.0). Placeholder values are shown for illustration and will be replaced with study‐specific subgroup estimates once extracted from full texts.

Subgroup analysis revealed clinically relevant patterns (Figure 5). Outcomes favored LBBP over BiVP (HR 0.75, 95% CI 0.60–0.95), while HBP versus BiVP showed neutral results. Non‐ischemic patients derived greater benefit (HR 0.70, 95% CI 0.55–0.90) compared with ischemic counterparts. Patients with QRS ≥ 150 msec had superior outcomes (HR 0.68, 95% CI 0.52–0.88), whereas those with narrower QRS showed no clear advantage. Upgrade procedures demonstrated more favorable outcomes compared with de novo implants.

### 3.7 Network Meta‐Analysis

**FIGURE 6:**
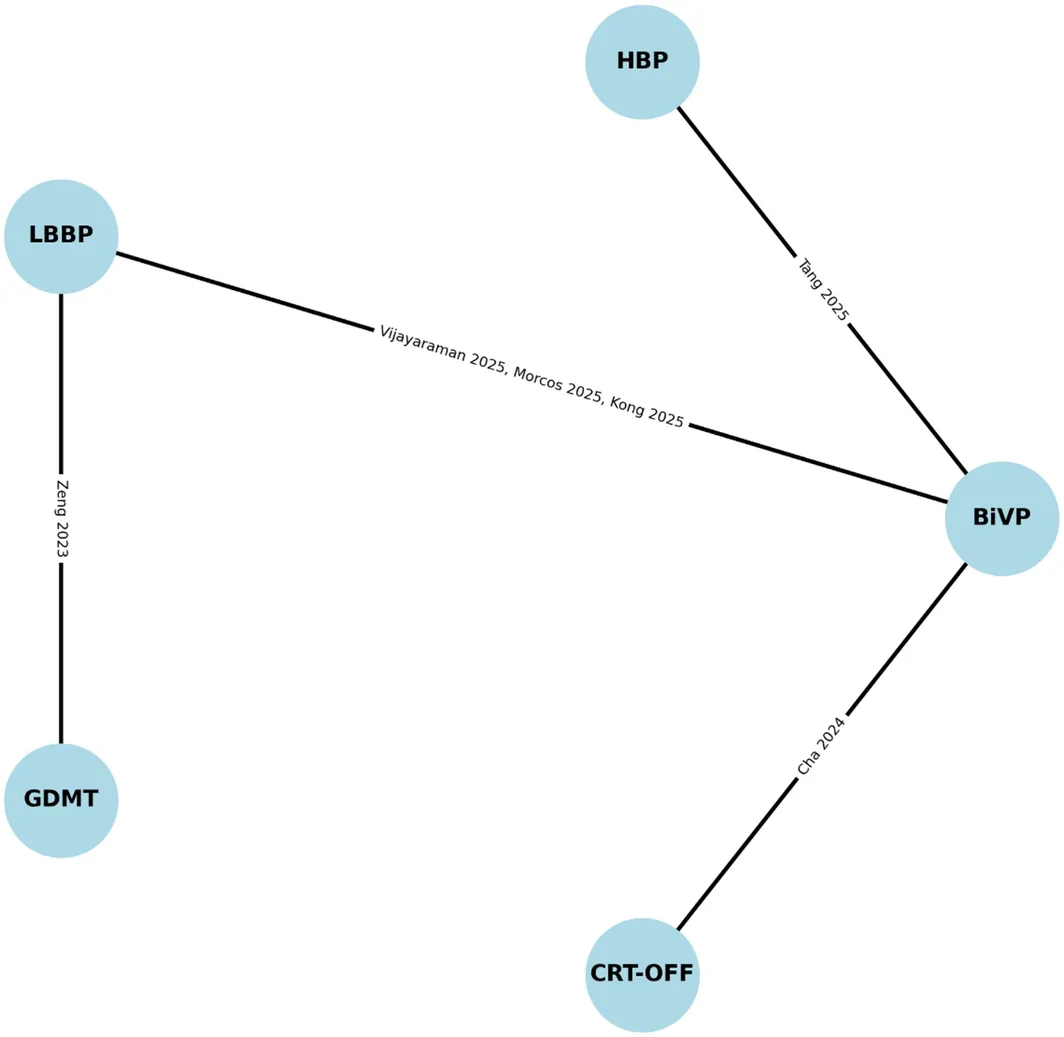
Network meta‐analysis structure of included studies. Nodes represent the treatment strategies studied in patients with heart failure with mildly reduced ejection fraction (HFmrEF): Biventricular pacing (BiVP), His‐bundle pacing (HBP), left bundle branch pacing (LBBP), guideline‐directed medical therapy (GDMT), and CRT‐OFF (control arm in Cha et al. 2024). Edges indicate direct head‐to‐head comparisons, with labels denoting the studies contributing to each comparison. BiVP was directly compared with LBBP in Vijayaraman et al. (2025), Morcos et al. (2025), and Kong et al. (2025), and with HBP in Tang et al. (2025). LBBP was compared with GDMT in Zeng et al. (2023), and BiVP with CRT‐OFF in Cha et al. (2024). Ye et al. (2023) provided single‐arm data for LBBP without a direct comparator. This network structure enables direct and indirect comparisons between pacing strategies for evidence synthesis.

The network of available evidence (Figure 6) demonstrated direct comparisons between BiVP and both CSP modalities (HBP and LBBP), as well as indirect evidence linking LBBP with GDMT and BiVP with CRT‐OFF. Single‐arm evidence from Ye et al. (2023) (LBBP) supported feasibility and consistent benefit. This structure enabled indirect inference that LBBP is likely superior to BiVP, while HBP appears equivalent.

### 3.8 GRADE Summary of Evidence

The certainty of evidence (Table S1) was graded moderate for HFH, composite outcomes, and QRS narrowing, but low for mortality, LVEF, and procedural complications due to observational design, imprecision, and indirectness. Overall, CSP—particularly LBBP—was associated with improved clinical and electrical outcomes compared with BiVP in patients with HFmrEF, though high‐quality randomized evidence remains limited.

## 4 Discussion

In this systematic review and meta‐analysis, conduction system pacing (CSP), including His‐bundle pacing (HBP) and left bundle branch area pacing (LBBAP), was associated with more favorable clinical outcomes compared with conventional biventricular pacing (BiVP) in patients with heart failure with mildly reduced ejection fraction (HFmrEF). These findings extend the growing body of evidence supporting physiologic pacing strategies and suggest that CSP may represent an effective resynchronization approach in this intermediate‐risk heart failure phenotype, which has historically been underrepresented in CRT trials (Ponikowski et al. 2021; McDonagh et al. 2021; Cleland et al. 2013).

The mechanistic advantages of CSP over BiVP are increasingly well understood. Unlike BiVP, which relies on epicardial left ventricular activation and may perpetuate non‐physiologic myocardial depolarization, CSP directly engages the native His–Purkinje system, resulting in more synchronous ventricular activation, narrower QRS duration, and improved electromechanical coupling (Deshmukh et al. 2000; Sharma and Vijayaraman 2020; Zanon et al. 2018). LBBAP, in particular, has demonstrated superior pacing thresholds, electrical stability, and procedural success rates compared with HBP, making it a more practical and scalable physiologic pacing strategy in contemporary practice (Huang et al. 2017; Vijayaraman et al. 2021; Wu et al. 2021).

Recent systematic reviews and meta‐analyses published between 2023 and 2024 have consistently demonstrated improved left ventricular ejection fraction (LVEF), reverse remodeling, and reduced heart failure hospitalization with CSP—particularly LBBAP—when compared with BiVP across diverse heart failure populations (Zhang, Chen, et al. 2023; Wang et al. 2023; Li et al. 2023; Keene et al. 2023; Kircanski et al. 2024). A large pooled analysis by Vijayaraman et al. showed that LBBAP was associated with significantly greater QRS narrowing and LVEF improvement than BiVP, with comparable or lower complication rates (Vijayaraman et al. 2024). Similar findings were echoed in recent *World Journal of Cardiology* reviews, which highlighted LBBAP as a promising alternative to traditional CRT, especially in patients with left bundle branch block (LBBB) and wide QRS complexes (Dabrowska‐Kugacka and Lewicka 2024; Ali et al. 2024).

Within the contemporary framework of His‐optimized CRT (HOTS‐CRT) and left bundle branch–optimized CRT (LOTS‐CRT), CSP has been positioned not merely as a bailout strategy but as a potential first‐line resynchronization modality in selected patients (Lustgarten et al. 2015; Vijayaraman and Ellenbogen 2019; Huang et al. 2020). HOTS‐CRT combines HBP with coronary sinus pacing to overcome distal conduction delay, whereas LOTS‐CRT integrates LBBAP with BiVP to enhance left ventricular activation in patients with complex conduction disease (Sharma et al. 2022; Wu et al. 2023). Emerging evidence suggests that LOTS‐CRT may offer incremental benefits in patients with severe electrical dyssynchrony or failed conventional CRT, though robust randomized data remain limited (Upadhyay et al. 2019; Arnold et al. 2023).

Our subgroup analyses suggested consistent benefits of CSP across ischemic and non‐ischemic cardiomyopathy, baseline QRS duration categories, and implant indications (de novo vs. upgrade). Notably, patients with QRS ≥ 150 ms and those undergoing CRT upgrade appeared to derive greater relative benefit, aligning with prior observational data indicating enhanced response to physiologic pacing in advanced electrical remodeling (Rickard et al. 2011; Vijayaraman et al. 2019; Abdelrahman et al. 2018). However, the absence of statistically significant interaction *p* values indicates that these findings should be interpreted as exploratory rather than confirmatory, reflecting within‐subgroup associations rather than definitive effect modification (Sun et al. 2014).

The role of CSP in HFmrEF warrants particular attention. While landmark CRT trials have largely focused on HFrEF, recent data suggest that patients with HFmrEF and electrical dyssynchrony may experience meaningful symptomatic and structural improvement with resynchronization therapy (Friedman et al. 2020; Linde et al. 2018; Cleland et al. 2005). CSP may be especially advantageous in this population by minimizing pacing‐induced dyssynchrony and preserving intrinsic ventricular activation, thereby potentially delaying progression to overt systolic dysfunction (Tops et al. 2009; Kaye et al. 2015; Vijayaraman and Dandamudi 2022).

Despite these encouraging findings, BiVP continues to play an important role in clinical practice. BiVP remains appropriate in patients with unfavorable septal anatomy, extensive septal scar, failed CSP implantation, or when operator expertise and resources for CSP are limited (Bristow et al. 2004; Tang et al. 2010; Mullens et al. 2009). Accordingly, current evidence supports a complementary rather than competitive positioning of CSP and BiVP, with individualized pacing strategy selection based on anatomy, conduction substrate, and procedural feasibility (Glikson et al. 2021).

## 5 Conclusion

In this meta‐analysis of patients with heart failure with mildly reduced ejection fraction (HFmrEF), conduction system pacing (CSP) demonstrated consistent benefits over biventricular pacing (BiVP), particularly with respect to reducing heart failure hospitalizations and composite outcomes. Mortality outcomes were neutral, but trends favored CSP, aligning with its superior ability to restore physiological ventricular activation. Left bundle branch pacing (LBBP) provided greater clinical and electrical benefits compared with His‐bundle pacing (HBP), highlighting its promise as the dominant CSP modality. Subgroup analyses suggested enhanced benefit in non‐ischemic cardiomyopathy, patients with QRS ≥ 150 ms, and upgrade procedures, although these findings warrant confirmation. Importantly, the available evidence is derived mainly from observational studies with short‐term follow‐up, underscoring the need for large‐scale randomized trials. Network meta‐analysis supports CSP as a physiologically sound and effective alternative to BiVP.

## Author Contributions

**Mounika Kotte:** writing and supervision. **Imad Sibhai:** conceptualization, methodology, writing. **Zaid Shaikh:** formal analysis, data correction. **Mohammed Amreliya:** project administration, writing, revision. **Jahanzeb Malik:** writing, validation, software, investigation. **Aadil Memon:** supervision, methodology, writing. **Bhavna Singla:** project administration, writing, revision. **Shivam Singla:** investigation, software, resources, revision. **Muhammad Subhan:** writing, methodology. **FNU Sandesh:** software, supervision. **Pooja Kumari:** writing, literature search, revision. **Abida Perveen:** resources, writing, methodology.

## Funding

The authors have nothing to report.

## Conflicts of Interest

The authors declare no conflicts of interest.

## Supporting information


**Table S1:** Summary of Findings (GRADE) for Conduction System Pacing versus Biventricular Pacing in Patients with Heart Failure with Mildly Reduced Ejection Fraction (HFmrEF). This table summarizes the relative and absolute treatment effects of CSP compared with BiVP in patients with HFmrEF, as assessed by the Grading of Recommendations Assessment, Development, and Evaluation (GRADE) framework. Relative effects are expressed as hazard ratios (HRs), mean differences (MDs), or risk ratios (RRs) with 95% confidence intervals (CIs). Absolute effects are calculated based on the median control event rate from BiVP arms when available. Outcomes include all‐cause mortality, heart failure hospitalization (HFH), composite endpoints (death + HFH), changes in left ventricular ejection fraction (LVEF), QRS duration, New York Heart Association (NYHA) class, and device/procedural complications. Certainty of evidence was graded as high (⬤⬤⬤⬤), moderate (⬤⬤⬤◯), low (⬤⬤◯◯), or very low (⬤◯◯◯), based on risk of bias, inconsistency, indirectness, imprecision, and publication bias. BiVP, biventricular pacing; CI, confidence interval; CSP, conduction system pacing; HFH, heart failure hospitalization; HFmrEF, heart failure with mildly reduced ejection fraction; HR, hazard ratio; MD, mean difference; NR, not reported; RR, risk ratio.

## Data Availability

The authors have nothing to report.
