# Supplementary material for: Conduction System Pacing Versus Biventricular Cardiac Resynchronization in HFmrEF: A Systematic Review and Meta‐Analysis
Source: Ann Noninvasive Electrocardiol. 2026 Mar 28;31(3):e70174. doi: 10.1111/anec.70174 (PMC13580865; doi:10.1111/anec.70174)
Supplement: Supplementary file 1 [file ANEC-31-e70174-s001.docx]

**Supplementary Table 1.**  Summary of Findings (GRADE) for Conduction System Pacing versus Biventricular Pacing in Patients with Heart Failure with Mildly Reduced Ejection Fraction (HFmrEF). This table summarizes the relative and absolute treatment effects of CSP compared with BiVP in patients with HFmrEF, as assessed by the Grading of Recommendations Assessment, Development, and Evaluation (GRADE) framework. Relative effects are expressed as hazard ratios (HRs), mean differences (MDs), or risk ratios (RRs) with 95% confidence intervals (CIs). Absolute effects are calculated based on the median control event rate from BiVP arms when available. Outcomes include all-cause mortality, heart failure hospitalization (HFH), composite endpoints (death + HFH), changes in left ventricular ejection fraction (LVEF), QRS duration, New York Heart Association (NYHA) class, and device/procedural complications. Certainty of evidence was graded as high (⬤⬤⬤⬤), moderate (⬤⬤⬤◯), low (⬤⬤◯◯), or very low (⬤◯◯◯), based on risk of bias, inconsistency, indirectness, imprecision, and publication bias. CSP = conduction system pacing; BiVP = biventricular pacing; HFmrEF = heart failure with mildly reduced ejection fraction; HFH = heart failure hospitalization; HR = hazard ratio; MD = mean difference; RR = risk ratio; CI = confidence interval; NR = not reported.

| **Outcome** | **Relative effect (95% CI)** | **Absolute effect (per 1,000)*** | **Participants (studies)** | **Certainty (GRADE)** | **Comments** |
| --- | --- | --- | --- | --- | --- |
| All-cause mortality | HR 0.82 (0.63–1.07) | Not calculated | ≤50% EF cohort incl. HFmrEF (1 large obs, PSM) | ⬤⬤◯◯ Low | Direction favors CSP but CI crosses 1; HFmrEF-only subgroup not isolated. Confounding likely (observational). |
| HF hospitalization | HR 0.63 (0.49–0.82) | Not calculated | ≤50% EF cohort incl. HFmrEF (1 large obs, PSM) | ⬤⬤⬤◯ Moderate | Consistent reduction with CSP; upgraded for magnitude/consistency, downgraded for non-randomized design. |
| Composite (death+HFH) | HR 0.81 (0.66–0.98) | **−122 per 1,000**† | ≤50% EF cohort incl. HFmrEF (1 large obs, PSM) | ⬤⬤⬤◯ Moderate | Driven mainly by HFH reduction; mortality neutral. |
| ΔLVEF (%) | Not estimable | — | HFmrEF comparative CSP vs BiVP data not extractable from abstracts | ⬤⬤◯◯ Low | Comparative HFmrEF arm-level means/SDs unavailable; single-arm signals (LBBP) positive but non-comparative. |
| QRS narrowing (ms) | MD −14 ms (CSP vs BiVP)** | — | ≤50% EF cohort incl. HFmrEF (1 large obs, PSM) | ⬤⬤⬤◯ Moderate | Paced QRS 129±19 (CSP) vs 143±22 (BiVP). Clinically meaningful electrical synchrony gain. |
| NYHA improvement | Not estimable | — | Comparative HFmrEF data not extractable | ⬤⬤◯◯ Low | Reported inconsistently; often single-arm. |
| Device/procedural complications | RR ≈0.54 (3.5% vs 6.5%) | Not calculated | ≤50% EF cohort incl. HFmrEF (1 large obs, PSM) | ⬤⬤◯◯ Low | Lower complications with CSP in aggregate; CI not available; non-randomized, potential reporting bias. |

* Absolute effects usually use the median control event rate from BiVP arms.

† Computed from HFmrEF I-CLAS composite control rate **34%** (BiVP 340/1000) and **HR 0.64**, yielding ≈218/1000 with CSP; **absolute risk reduction ~122/1000** (12.2%).
